# Anti-infective compounds for *Yersinia*

**DOI:** 10.1093/sumbio/qvag014

**Published:** 2026-04-11

**Authors:** Kumar D Gahlot, Matthew S Francis, Jyoti M Gurung

**Affiliations:** Department of Molecular Biology, and Umeå Centre for Microbial Research, Umeå University, SE-90187 Umeå, Sweden; Department of Molecular Biology, and Umeå Centre for Microbial Research, Umeå University, SE-90187 Umeå, Sweden; Department of Molecular Biology, and Umeå Centre for Microbial Research, Umeå University, SE-90187 Umeå, Sweden

**Keywords:** *Yersinia* infections, virulence factors, traditional antibiotic therapies, novel anti-bacterial strategies, vaccine development

## Abstract

*Yersinia* is a genus of bacteria that includes several species known to cause diseases in humans and animals. We explore developments in anti-bacterial compounds to counteract *Yersinia* infections, focusing on both traditional antibiotic therapies and novel strategies targeting virulence factors, host-pathogen interactions, and vaccine development. This review evaluates current strategies for the treatment and management of *Yersinia* infections, outlining key challenges and emerging opportunities for improved clinical outcomes.

**Sustainability statement**

Much of this article aligns with the Sustainable Development Goal (SDG) 3: Good Health and Well-being, as *Yersinia*’s environmental resilience and zoonotic transmission promote acquisition of resistance genes, increasing the threat of multidrug-resistant outbreaks. Developing alternative anti-*Yersinia* therapeutics advances SDG 3 by lowering infection burdens, preserving antibiotic effectiveness, and strengthening publichealth preparedness. These efforts have relevance to other SDGs—protecting agriculture and ecosystems (SDG 14: Life Below Water; SDG 15: Life on Land), improving food security and sustainable production (SDG 2: Zero Hunger; SDG 12: Responsible Consumption and Production), reducing dualuse risks (SDG 16: Peace, Justice and Strong Institutions), and mitigating climate-driven pathogen evolution (SDG 13: Climate Action).

## *Yersinia* species as models for evolutionary, environmental, and infection biology

### An introduction to known *Yersinia* spp.

The genus *Yersinia* comprises over 25 species of Gram-negative, facultatively anaerobic bacilli within the family *Yersiniaceae* (Adeolu et al. 2016, Kingry et al. 2023). Among these, *Yersinia pestis, Y. enterocolitica*, and *Y. pseudotuberculosis* are the most clinically relevant. *Y. pestis* is a zoonotic pathogen and causes three primary forms of disease in humans: bubonic, septicaemic, and pneumonic plague (Barbieri et al. 2021). Bubonic plague, the most common form, is characterised by swollen lymph nodes (buboes), while septicaemic plague involves bloodstream infection, and pneumonic plague affects the lungs and can spread person-to-person. Prompt antibiotic treatment is essential, as all forms can be fatal if untreated. *Y. enterocolitica* and *Y. pseudotuberculosis* are also zoonotic pathogens that infect a wide range of animals (Galindo et al. 2011). Pigs are the principal reservoir for *Y. enterocolitica*, while *Y. pseudotuberculosis* is found in various wild and domestic animals, especially rodents and birds. Humans are incidental hosts, typically acquiring infection through contaminated food, water, or contact with infected animals. *Yersinia ruckeri* is a well-established veterinary pathogen of aquatic animals, particularly salmonids (Kumar et al. 2015), while *Y. entomophaga* is associated with insect pathogenicity and also holds potential for biotechnological applications (Hurst et al. 2015, Hurst et al. 2020). In addition, the presence of known virulence determinants in the genome of *Y. wautersii* suggests that this species may also possess pathogenic potential (Savin et al. 2014). In contrast, the remaining species—*Y. aldovae, Y. aleksiciae, Y. alsatica, Y. artesiana, Y. bercovieri, Y. canariae, Y. frederiksenii, Y. hibernica, Y. intermedia, Y. kristensenii, Y. massiliensis, Y. mollaretii, Y. nurmii, Y. occitanica* (alternative synonym; *Y. rochesterensis*)*, Y. pekkanenii, Y. proxima, Y. rohdei, Y. similis, Y. thracica*, and *Y. vastinensis*—are generally regarded as non-pathogenic or opportunistic, although they are occasionally isolated from human or animal specimens (Sulakvelidze 2000, Chen et al. 2010, Le Guern et al. 2020, Kislichkina et al. 2024). Together, these species illustrate the considerable taxonomic and ecological breadth of the *Yersinia* genus, spanning highly adapted pathogens, environmental isolates, and opportunistic organisms of varying clinical and agricultural relevance.

### Clinical manifestations of the enteric *Yersinia*

Yersiniosis is a zoonotic disease complex caused by several species within the genus *Yersinia*, notably *Y. enterocolitica, Y. pseudotuberculosis*, and, in aquatic animals, *Y. ruckeri*. Each species exhibits distinct ecological niches, virulence mechanisms, and clinical manifestations (Tauxe 2004, Galindo et al. 2011, Kumar et al. 2015, Shoaib et al. 2019, Grygiel-Gorniak 2025).

*Y. ruckeri* is a globally distributed aquaculture pathogen that causes enteric redmouth disease, with higher infection and disease impact in countries with intensive salmonid farming, particularly Norway, Chile, Scotland, Canada, and the Faroe Islands, where significant economic losses are consistently reported and can lead to flow-on disruptions in food supply (Kumar et al. 2015). The pathogen poses the greatest risk to highly susceptible salmonids, especially rainbow trout (*Oncorhynchus mykiss*) and Atlantic salmon (*Salmo salar*), which dominate production in these high-risk regions. This motile bacterium is characterised by its ability to produce extracellular enzymes such as proteases and haemolysins, contributing to tissue damage in infected hosts (Guijarro et al. 2018, Wrobel et al. 2019). The bacterium thrives in aquatic environments, especially under intensive aquaculture conditions, and exhibits clonal diversity with specific lineages associated with outbreaks in different geographic regions (Haig et al. 2011, Barnes et al. 2016, Gulla et al. 2018, Riborg et al. 2023).

In contrast, *Y. enterocolitica* is a significant human pathogen. It is commonly transmitted through contaminated food, particularly undercooked pork, and can proliferate at refrigeration temperatures due to its psychrotrophic nature (Shoaib et al. 2019, Grygiel-Gorniak 2025). Pathogenic strains are typically classified into biotypes 1B and 2–5, with serotype O:3 being predominant in Europe. These strains harbour a virulence plasmid (pYV) encoding a type III secretion system (T3SS) and adhesins such as YadA, which facilitate host cell invasion and immune evasion. Clinically, *Y. enterocolitica* infection presents as gastroenteritis, mesenteric lymphadenitis, and can mimic appendicitis (Naktin and Beavis 1999, Galindo et al. 2011, Shoaib et al. 2019, Grygiel-Gorniak 2025). Post-infectious sequelae such as reactive arthritis are also documented, particularly in HLA-B27-positive individuals (Naktin and Beavis 1999).

*Y. pseudotuberculosis* is another enteropathogen affecting both humans and animals. It is environmentally resilient, persisting in soil and water, and is frequently isolated from wild animals, especially rodents and birds (Naktin and Beavis 1999, Tauxe 2004, Galindo et al. 2011, Grygiel-Gorniak 2025). Like *Y. enterocolitica*, it possesses a virulence plasmid and shares many pathogenic mechanisms. Infections typically result in fever, abdominal pain, and mesenteric lymphadenitis (Naktin and Beavis 1999, Tauxe 2004, Galindo et al. 2011, Grygiel-Gorniak 2025). However, a distinct clinical entity known as Far East scarlet-like fever has been described in Russia and Japan (Amphlett 2016, Suntsov 2025). This syndrome is characterised by high fever, diffuse erythematous rash, desquamation, and systemic inflammation, resembling classical scarlet fever. It is associated with specific clonal lineages of *Y. pseudotuberculosis* and is often linked to ingestion of contaminated vegetables or untreated water (Amphlett 2016, Suntsov 2025).

### Evolution of *Y. pestis* and climate-change-driven pathogenesis

*Y. pestis*, derived from the enteropathogen *Y. pseudotuberculosis*, has undergone significant genomic remodelling over the past 20 000–30 000 years (Wu et al. 2022, Suntsov 2025). This transformation involved the loss of genes associated with enteric colonisation and the acquisition of plasmids, enabling flea-borne, systemic transmission, which converted it into one of the most virulent pathogens known. Its persistence in nature is maintained through a sylvatic cycle involving wild rodents and flea vectors (Wimsatt and Biggins 2009, Barbieri et al. 2021). Under stable ecological conditions, *Y. pestis* circulates at low levels. However, environmental disruptions such as climate change, habitat degradation, or food scarcity can trigger rodent population crashes, prompting infected fleas to seek alternative hosts, including humans, thereby facilitating zoonotic spillover (Wimsatt and Biggins 2009, Barbieri et al. 2021).

Contemporary genomic surveillance reveals ongoing evolution (Wu et al. 2022, Suntsov 2025). Phylogenomic analyses have identified geographically distinct lineages, evolutionary bottlenecks, and horizontal gene transfers that shape virulence and adaptability. Key virulence factors include the antiphagocytic F1 capsule (encoded on pMT1/pFraI), plasminogen activator Pla (encoded on pPCP1), and a specialised T3SS (encoded on pCD1), all of which contribute to immune evasion and systemic infection. The evolutionary trajectory of *Y. pestis* exemplifies how genomic plasticity and ecological pressures drive the emergence of highly pathogenic organisms.

Recent studies have further illuminated the role of climate change as a powerful evolutionary force in the emergence and adaptation of *Y. pestis*. Suntsov proposed that the speciation of *Y. pestis* from *Y. pseudotuberculosis* was triggered by climatic shifts in Central Asia during the late Pleistocene (Suntsov 2022). The aridification of the Gobi region and the Sartan glaciation (22 000–15 000 years ago) induced behavioural changes in Tarbagan marmots (*Marmota sibirica*) and their fleas (*Oropsylla silantiewi*), creating novel ecological conditions that enabled a traumatic, non-alimentary infection route during hibernation. This facilitated the transition of a *pseudotuberculosis* clone into a blood-borne pathogen, exemplifying how climate-induced behavioural plasticity in host-vector systems can catalyse microbial speciation.

Cui and colleagues provided genomic evidence that *Y. pestis* continues to evolve in response to environmental variability (Cui et al. 2020). In a 40-year longitudinal study of a natural plague focus in the Tien Shan mountains, researchers identified strong positive selection on *rpoZ*, which encodes the omega (ω) subunit of DNA-directed RNA polymerase, a structural element that stabilizes the enzyme and supports proper assembly of its core subunits. Positive selection on *rpoZ* likely enhances RNA polymerase conformation in ways that modulate transcriptional programs linked to biofilm production, a key trait for efficient flea-borne transmission. Variants of *rpoZ* associated with increased biofilm-forming capacity emerged during colder, drier periods, suggesting that climate-driven selective pressures shape transmission strategies through extended phenotypes. These findings underscore the dynamic interplay between climate, vector behaviour, and bacterial adaptation.

Eaton and colleagues contextualised these findings within a global phylogenetic framework (Eaton et al. 2023). Analysing over 600 *Y. pestis* genomes, they demonstrated that the pathogen’s evolutionary rate is highly variable and often decoupled from historical outbreak timelines. Their work revealed that pandemic lineages may have emerged centuries before their documented appearance, and that climatic and ecological factors likely contributed to their cryptic persistence and episodic reemergence. Importantly, they caution that genetic data alone may be insufficient to reconstruct short-term epidemic dynamics, and instead advocate for integrative approaches that combine genomic, ecological, and historical data.

These findings align with foundational work by Stenseth and coworkers, who demonstrated that plague dynamics in Central Asia are strongly influenced by climate variation, particularly temperature and precipitation, which affect rodent and flea populations and thus the risk of epizootics (Stenseth et al. 2006). Xu and colleagues further showed that wet climate and transportation routes accelerated the global spread of plague during the Third Pandemic (Xu et al. 2014), reinforcing the importance of environmental and anthropogenic factors in shaping disease emergence.

**Figure 1:**
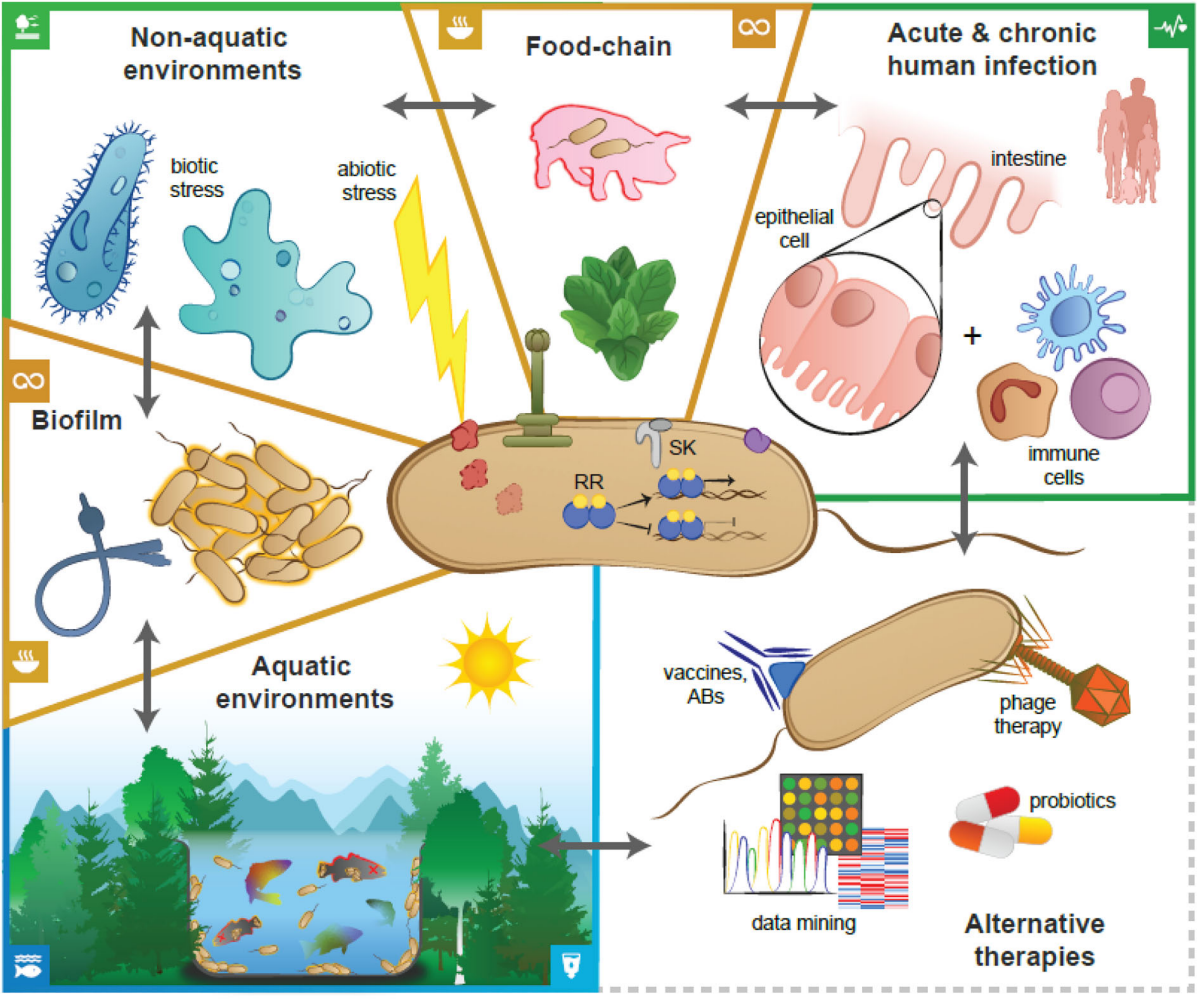
Conceptual overview of *Yersinia* infection, ecology, therapeutic innovations, and sustainability context. The figure depicts the ecological and epidemiological dimensions of *Yersinia* infections, including human and fish hosts, environmental reservoirs (aquatic and terrestrial), and food-chain transmission routes. It highlights bacterial survival strategies including biofilm formation and responses to biotic and abiotic stress, as well as host-pathogen interactions involving intestinal epithelial and immune cells. Emerging antibacterial interventions are shown, including traditional antibiotics, phage therapy, vaccines, monoclonal antibodies, probiotics, and data-driven approaches for drug discovery. This framework is aligned with key United Nations Sustainable Development Goals: Zero Hunger and Responsible Consumption and Production (by reducing foodborne infections and ensuring safe food systems), Good Health and Well-being (by mitigating human morbidity and mortality), Clean Water and Sanitation (through control of aquatic reservoirs), Life on Land and Life Below Water (by protecting terrestrial and aquatic ecosystems from pathogen spillover), and Peace, Justice and Strong Institutions (by reducing the risk of *Yersinia* species being exploited as dual-use agents). Together, these strategies support global health, food security, environmental sustainability, and biosecurity. (Produced by DC SciArt, Umeå, Sweden)

This body of evidence is directly relevant to the United Nations Sustainable Development Goals (SDGs). The link between climate and pathogen evolution underscores the urgency of SDG 13 (Climate Action), as global warming and altered precipitation regimes may reshape the distribution and behaviour of rodent hosts and flea vectors, potentially reactivating dormant plague foci or creating new ones. This poses a renewed threat to SDG 3 (Good Health and Well-being), particularly in regions with limited public health infrastructure. Moreover, the ecological specificity of plague reservoirs, such as the marmot–flea system, highlights the importance of SDG 15 (Life on Land). Protecting biodiversity and maintaining ecosystem integrity are essential not only for conservation but also for buffering zoonotic spillovers (Fig. 1).

In sum, the evolutionary plasticity of *Y. pestis* serves as a cautionary example of how environmental change can reshape pathogen landscapes. As anthropogenic climate change accelerates, it is imperative to monitor how these shifts influence the ecology and evolution of zoonotic pathogens. Strengthening interdisciplinary research that bridges genomics, ecology, and climate science is essential for building resilient health systems and achieving long-term sustainability goals (Fig. 1).

### Present clinical challenges

According to the European Centre for Disease Prevention and Control (ECDC), 8 037 confirmed cases of yersiniosis were reported in the EU/EEA in 2022, with a notification rate of 2.2 per 100 000 population European Food Safety Authority (EFSA) (2023). Most cases were attributed to *Y. enterocolitica*, with serogroup O:3 being the most prevalent. Hospitalisation occurred in approximately 35–40% of cases, while mortality remained low, with fewer than 10 deaths annually. The trend over the past five years has been relatively stable, though some countries have reported slight increases, likely due to improved diagnostic capabilities and reporting systems European Food Safety Authority (EFSA) (2023). The Centers for Disease Control and Prevention (CDC) reports that 200–300 laboratory-confirmed cases of yersiniosis occur annually in the U.S.A, with a hospitalisation rate of about 30% (Delahoy et al. 2023). Again, mortality is extremely rare, and most infections were self-limiting. Globally, yersiniosis is not among the top infectious disease burdens tracked by the World Health Organization (WHO), reflecting its low global mortality impact compared to other enteric diseases.

While yersiniosis remains a low-mortality disease, its morbidity burden, especially in terms of hospitalisations and severe post-infectious complications like reactive arthritis in at-risk populations, warrants continued surveillance. Ongoing improvements in food safety, public awareness, and diagnostic practices are essential to maintaining control over its spread (Grygiel-Gorniak 2025). Moreover, the prevalence of *Yersinia* species in the environment means that it is an important contributor to the rapid dissemination of antimicrobial resistance (AMR).

The growing global threat of AMR is driven by factors such as antibiotic overuse, slow commercial drug development, socio-economic disparities, and climate change (MacFadden et al. 2018, Lambraki et al. 2022, Darby et al. 2023, Walsh et al. 2023, El-Maradny et al. 2025). In 2019 alone, antibiotic-resistant infections were linked to over 4.95 million deaths globally (Antimicrobial Resistance Collaborators 2022). This is also reflected in clinical manifestations caused by pathogenic *Yersinia* species. *Y. pestis*, the plague agent, has reemerged in regions across Eurasia, Africa, and the Americas, with Madagascar accounting for 80% of global cases between 2013–2018 (Randremanana et al. 2019, Barbieri et al. 2021, Rakotosamimanana et al. 2021). Although treatable, resistant *Y. pestis* strains have been identified, including 16/95 and 17/95, which carry transferable plasmids conferring resistance to streptomycin, tetracyclines, and sulfonamides (Guiyoule et al. 2001, Galimand et al. 2006, Andrianaivoarimanana et al. 2022, Lei and Kumar 2022). This is a critical concern given that streptomycin and tetracycline are classic first-line drugs for plague treatment (*Galimand* et al. *2006*). Hence, the emergence of AMR among recent plague outbreaks has prompted the WHO to establish thorough guidelines for vigilant surveillance of *Y. pestis* (Bertherat and Jullien 2024).

Similarly, multidrug-resistant (MDR) *Y. enterocolitica* and *Y. pseudotuberculosis* have been isolated from food and environmental sources, causing outbreaks linked to pork, unpasteurised milk, and vegetables (Tavassoli et al. 2018, Shoaib et al. 2019, Karlsson et al. 2021, Huang et al. 2023). These MDR strains exhibit resistance particularly to β-lactams (penicillin, ampicillin, first-generation cephalosporins) and in some cases fluoroquinolones, due to mechanisms such as chromosomally encoded β-lactamases and *gyrA* mutations. While most yersiniosis cases are mild, severe infections require antimicrobial therapy; fortunately, many clinical isolates remain susceptible to trimethoprim-sulfamethoxazole, third-generation cephalosporins, fluoroquinolones, tetracyclines, and aminoglycosides, meaning that MDR strains currently pose limited risk to treatment efficacy. However, the detection of *Y. enterocolitica* 4/O:3 and *Y. pseudotuberculosis* strains carrying transferable resistance elements (Karlsson et al. 2021, Huang et al. 2023), raises concern for environmental AMR exchange, particularly in agricultural settings where antimicrobial use can select for resistant clones. Given their environmental resilience and zoonotic potential, *Yersinia* species are well-equipped to acquire and transfer novel genetic elements, increasing the risk that MDR strains could intensify future outbreaks and complicate public-health responses. Therefore, sustainable strategies to mitigate AMR in *Yersinia* are critically needed (Fig. 1).

## Anti-infective strategies for combatting *Yersinia* infections

A promising alternative to conventional antibiotics is the use of anti-infective strategies, which disarm pathogens by targeting virulence factors that would normally enable immune evasion or host damage. Unlike antibiotics that inhibit essential bacterial processes and exert strong selective pressure for resistance, anti-infective agents neutralise specific pathogenic mechanisms without affecting commensal bacteria. This targeted approach reduces selective pressure and may slow the emergence of resistance (Keyser et al. 2008, Duncan et al. 2012, Dickey et al. 2017). Among the most studied targets are adhesins, toxins, and bacterial appendages, with the T3SS emerging as a particularly attractive candidate (Keyser et al. 2008, Fasciano et al. 2019). This section explores the evolving paradigm of virulence blockers as sustainable alternatives to antibiotics using pathogenic *Yersinia* as a model, discussing their development, potential, and limitations. While T3SS inhibitors have been extensively reviewed, here we include other targets such as the CpxA/CpxR two-component system, phage therapy, and *Yersinia*-derived immune modulators and adjuvants (summarised in Table 1). Additionally, the virulence mechanisms of *Y. ruckeri*, a significant pathogen in aquaculture, are examined alongside current mitigation strategies, offering a broader perspective on anti-infective approaches (Table 1).

**Table 1:** Anti-infective strategies against *Yersinia* and related pathogens.

Strategy/compound	Target/mechanism	Host/pathogen	Reference
Anti-*Yersinia* virulence blockers			
Salicylidene Acylhydrazides (SAHs) including INP0007 & INP0010	T3SS assembly inhibition	*Y. pseudotuberculosis*	(Kauppi et al. 2003, Nordfelth et al. 2005)
Benzimidazole compounds	T3SS transcriptional regulation (LcrF)	*Y. pestis*	(Kim et al. 2009, Garrity-Ryan et al. 2010)
Piericidin A1	T3SS needle assembly	*Y. pseudotuberculosis*	(Duncan et al. 2014)
MBX 1641 (Phenoxyacetamide)	Yop effector secretion	*Y. pestis*	(Aiello et al. 2010)
Cyclic peptomers (EpD-3′N, 4EpDN)	Needle stability	*Y. pseudotuberculosis*	(Lam et al. 2017, Lam et al. 2021)
p-Nitrocatechol sulfate (pNCS)	YopH phosphatase active site	*Y. pestis*	(Sun et al. 2003)
Nitro-containing small molecules	Activates CpxR∼P-dependent transcription by inhibiting CpxA phosphatase	Uropathogenic *E. coli*	(van Rensburg et al. 2015)
CpxA phosphatase inhibitor	Locks Cpx system in active state, represses virulence genes	Uropathogenic *E. coli*	(Fortney et al. 2022)
Furanones & analogues	Quorum sensing (LuxIR system)	*Y. enterocolitica*	(Rasmussen et al. 2005)
Vanillic acid	AHL signaling inhibition	*Y. enterocolitica*	(Sivasankar et al. 2020)
Triphenylbismuth dichloride	Pyruvate dehydrogenase (central metabolism)	Several pathogens	(Birkenstock et al. 2012)
Iron chelators/siderophore conjugates	Iron acquisition systems (Yersiniabactin)	*Y. pestis*	(Rakin et al. 2012, Rayner et al. 2023)
Fatty acid biosynthesis inhibitors	Lipid biosynthesis enzymes	Several Gram-negative pathogens	(Carter et al. 2025)
ABC transporter blockers	Nutrient uptake systems	*Enterobacteriaceae*	(Akhtar and Turner 2022)
Amino acid biosynthesis inhibitors	Carbon metabolism pathways	Multiple pathogens	(Tong and Brown 2023)
Host-directed therapies			
Autophagy inducers	Enhance phagolysosome maturation	Host macrophages infected by *Yersinia*	(Deuretzbacher et al. 2009, Alem et al. 2015)
Immune checkpoint inhibitors	Restore T-cell effector function	Host immune system	(Henriksen et al. 2025)
Macrophage polarization agents	Promote M1 phenotype, enhance NO and cytokine release	Host macrophages	(Bergsbaken and Cookson 2007)
Cytokine modulators	TNF–IL-1 axis activation	Host intestinal immune cells	(Matsuda et al. 2024)
Nutritional immunity enhancers	Iron sequestration (hepcidin, ferritin)	Host systemic response	(Murdoch and Skaar 2022)
Vaccine design			
rF1V fusion protein	F1 capsule + LcrV antigen	*Y. pestis*	(Powell et al. 2005, Chichester et al. 2009)
Live attenuated strains	Δ*pla*, Δ*caf*1, Δ*lcr*V mutants	*Y. pestis*	(Sun et al. 2011, Derbise et al. 2020)
Vector-based platforms	Recombinant *Francisella* LVS expressing F1 & LcrV	*Y. pestis*	(Jia et al. 2025)
YopB/YopD subunit	Conserved translocon proteins	*Yersinia* spp.	(Heine et al. 2019)
Oral/immersion fish vaccines	Outer membrane proteins (OmpA, OmpF)	*Y. ruckeri*	(Ormsby et al. 2019, Yang et al. 2021)
Probiotics			
Probiotic microbes and components added to feeds to enhance fish health	Fish microbiome health	*Y. ruckeri*	(Raida et al. 2003, Abbass et al. 2010)

### The type III secretion injectisome

Rosqvist and colleagues first proposed a dedicated nanomachine in pathogenic *Yersinia* capable of delivering bacterial proteins into eukaryotic cells that is structurally and evolutionarily related to the flagellum (Rosqvist et al. 1994). This T3SS apparatus is now recognised as a conserved virulence mechanism across diverse Gram-negative pathogens. Functional T3SSs are often plasmid- or pathogenicity island-encoded, and their disruption leads to attenuated virulence in animal models. In *Yersinia*, the plasmid-encoded variant is known as the Ysc-Yop T3SS (Cornelis et al. 1998, Plano and Schesser 2013).

Structurally, the T3SS resembles a molecular syringe. Its basal body, composed of ∼12 Sct proteins, spans the bacterial membranes. A needle formed by SctF polymers extends extracellularly, capped by a translocon complex (SctA, SctB, SctE) that forms pores in host membranes (Kubori et al. 1998, Hu et al. 2017, Hu et al. 2018). On the cytoplasmic side, the C-ring and SctN ATPase complex form the sorting platform and coordinate substrate recognition and secretion. Effector proteins, or Yops in *Yersinia*, are targeted to the base by chaperones for ordered secretion (Akeda and Galan 2005, Lara-Tejero et al. 2011, Gurung et al. 2022, Wimmi et al. 2024). Upon host contact, YopH, YopE, YopM, YopP/J, and YpkA/YopO effector proteins are translocated into host cells, where they modulate immune responses by inhibiting phagocytosis, suppressing inflammatory signalling, and altering cell death pathways to facilitate bacterial colonisation (Grabowski et al. 2017, Mecsas 2019, Schubert et al. 2020). Specifically, YopH acts as a tyrosine phosphatase that disrupts early phagocytic signalling, while YopE inactivates Rho GTPases to destabilize the actin cytoskeleton, further impairing phagocytosis. YopM modulates intracellular immune signalling by activating host kinases RSK1 and PRK2, whereas YopP/J inhibits NF-κB and MAPK pathways, blocking cytokine responses and promoting immune suppression. YpkA/YopO, a serine/threonine kinase that binds actin and Rho GTPases, disrupts cytoskeletal dynamics and interferes with additional host signalling required for effective phagocytic function.

This mechanistic understanding has spurred the development of anti-infective agents targeting various aspects of the T3SS, including its transcription, assembly, and effector functions. Current strategies encompass high-throughput screening (HTS), computational modelling, and structure-based drug design. Here, we highlight key studies that illustrate both the promise and the challenges of T3SS-targeted anti-infective approaches, and advocate for integrated methodologies that combine phenotypic and target-based screening.

#### Targeting T3SS structural components

HTS has played a pivotal role in the discovery of small-molecule inhibitors targeting the T3SS. A seminal study from Umeå University, Sweden employed a whole-cell HTS approach based on T3SS regulation in *Y. pseudotuberculosis*, utilising a luciferase reporter driven by the *yopE* promoter (Kauppi et al. 2003). Screening approximately 9 400 compounds, this assay identified Salicylidene Acylhydrazides (SAHs; C1–C3) and a sulfonamidobenzamide (SAB; C4) as T3SS inhibitors. Although initially thought to act *via* transcriptional repression, the precise molecular targets of these compounds remain undefined. Further investigations revealed more potent SAHs, including INP0007 and INP0010, which exhibited distinct effects on T3SS assembly (Kauppi et al. 2003, Nordfelth et al. 2005). INP0007 disrupted the formation of SctD and the needle complex, whereas INP0010 selectively inhibited needle assembly without affecting the basal body. However, whether these phenotypes result from direct target engagement or indirect interference remains unresolved (Morgan et al. 2018). Despite promising *in vitro* efficacy, off-target effects on bacterial viability and host cell integrity have impeded clinical translation.

In parallel with phenotypic screening, rational design strategies have been employed to target specific T3SS components. Swietnicki and colleagues conducted computational screening against the ATPase SctN of *Y. pestis*, identifying compounds 7086, 7812, and 7832 that inhibited ATPase activity and suppressed YopE secretion (Swietnicki et al. 2011). Nevertheless, these compounds failed to prevent cytopathic effects in HeLa cells, underscoring the challenges in translating *in vitro* inhibition into functional cellular outcomes.

#### HTS for anti-host effector agonists

Yop effector delivery into host immune cells is contact-dependent and requires a multi-component apparatus (basal body, needle, translocon), with polarised effector injection at the host interface (Rosqvist et al. 1994). A widely adopted method for monitoring effector delivery into host cells employs translational fusions between bacterial effectors and the TEM-1 β-lactamase reporter. Upon infection, successful delivery is indicated by a fluorescence shift from green to blue in the presence of a β-lactamase substrate, allowing real-time, quantitative analysis (Charpentier and Oswald 2004). This assay has been instrumental in identifying small-molecule inhibitors of the *Yersinia* T3SS, particularly those affecting Yop translocation. Using this system, HTS identified six compounds (C15, C19, C20, C22, C24, C38) that selectively inhibited polarised Yop delivery without disrupting T3SS assembly or general secretion, suggesting interference with host contact or translocon formation. Notably, C20 impaired bacterial adherence to mammalian cells, a prerequisite for efficient effector delivery (Harmon et al. 2010, Morgan et al. 2018).

Additional inhibitors, including MBX 1641 (phenoxyacetamide) and compound 3 (malic diamide), suppressed YopE secretion and T3SS activity in *Y. pestis* without affecting basal body or needle formation (Aiello et al. 2010, Morgan et al. 2018). Furthermore, screening based on the low-calcium response (LCR) phenotype identified compounds 1, 3, and 4, which restored bacterial growth under calcium-depleted conditions and reduced secretion of YopE, YopD, and YopM, protecting HeLa cells from T3SS-mediated cytotoxicity (Pan et al. 2009, Duncan et al. 2012). Moreover, synthetic cyclic peptomers, analogues of phepropeptin D, such as EpD-3′N and its stereochemically optimised derivative 4EpDN, inhibited YopE secretion and YopM delivery, likely by impairing needle assembly or stability in *Y. pseudotuberculosis* (Lam et al. 2017, Lam et al. 2021).

Other screens have focused on host cell responsiveness to T3SS-induced signalling upon *Yersinia* contact. *Y. pseudotuberculosis* activates NF-κB via its translocator proteins. Using HEK293T cells with an NF-κB-inducible luciferase reporter, piericidins A1 and Mer-A 2026B were identified as inhibitors of NF-κB activation and YopM translocation, without affecting bacterial viability. Piericidin A1 disrupted SctF needle assembly while preserving the basal body (Auerbuch et al. 2009, Duncan et al. 2014, Charro and Mota 2015, Morgan et al. 2017).

#### Structure-based approaches to identify inhibitors of anti-host effector activity

Structure-guided approaches, which integrate high-resolution protein structural data with virtual screening techniques, represent a strategic and targeted methodology for the discovery of T3SS inhibitors. In contrast to HTS, these methods enable a more focused interrogation of protein–protein interaction hotspots and support rational drug design, thereby potentially expediting the identification of lead compounds.

YopH, a pivotal T3SS effector and a highly active protein tyrosine phosphatase (PTP), plays a critical role in the virulence of *Yersinia* species. Its conserved phosphotyrosine (pTyr) binding pocket has been effectively exploited for the development of selective inhibitors. One such compound, *p*-nitrocatechol sulfate (pNCS), was identified as a selective YopH inhibitor with minimal activity against mammalian PTPs. Structural elucidation confirmed that pNCS binds directly within the YopH active site (Sun et al. 2003), facilitating a virtual screening campaign of over 400 000 compounds. This effort led to the identification of 14 structurally diverse inhibitors (Hu et al. 2013). Although *in vivo* efficacy was not evaluated, the study established a robust framework for anti-virulence drug discovery.

Subsequent screening of salicylate-based compounds bearing furanyl moieties yielded potent YopH inhibitors capable of reversing its immunosuppressive effects on T cell activation (Tautz et al. 2005). Nuclear magnetic resonance (NMR)-guided fragment-based screening further identified bidentate furanyl-salicylate derivatives with inhibitory concentrations in the nanomolar to micromolar range. Notably, compounds 2 and 6 demonstrated protective effects in HeLa cells against *Yersinia*-induced cytotoxicity (Vazquez et al. 2007, Leone et al. 2010).

#### Inhibition of other T3SS associated targets

Anti-virulence strategies targeting the T3SS have included interference with transcriptional regulation. In pathogenic *Yersinia* species, the AraC-type transcriptional activator LcrF functions as the master regulator of T3SS gene expression in response to environmental cues such as temperature (Schwiesow et al. 2015). A target-based screen against LcrF’s DNA-binding activity identified N-hydroxybenzimidazoles as effective anti-virulence agents, with some compounds conferring protection against *Y. pseudotuberculosis* in mammalian models (Kim et al. 2009, Garrity-Ryan et al. 2010). Additionally, the haloid-containing sulfonamidobenzamide compound C4, identified via a whole-cell reporter assay, was shown to inhibit LcrF promoter activity (Kauppi et al. 2003). However, subsequent studies questioned this mechanism, although C4 did disrupt T3SS function, as evidenced by the absence of SctF needle formation (Morgan et al. 2018).

Therapeutic antibodies targeting T3SS components represent another promising approach. As early as 1963, passive transfer of anti-V antigen antisera was shown to protect mice from *Y. pestis* infection (Lawton et al. 1963). The V antigen was later identified as LcrV (SctA), a critical T3SS component located at the needle tip (Mueller et al. 2005). Numerous murine monoclonal antibodies against LcrV, particularly mAb 7.3, have demonstrated protective efficacy against bubonic and pneumonic plague in mice. These antibodies bind LcrV, opsonise *Yersinia*, and inhibit Yop translocation and cytotoxicity (Cowan et al. 2005, Ivanov et al. 2014, Hotinger and May 2020). However, LcrV exhibits significant genetic variability, especially in regions encoding protective epitopes. For example, while anti-V antigen from *Y. pseudotuberculosis* conferred protection against plague, it was ineffective against *Y. enterocolitica*, highlighting cross-protection limitations (Motin et al. 1994, Daniel et al. 2019). To overcome this, alternative conserved antigens have been explored. Ivanov and colleagues investigated the translocon components YopB and YopD (SctE and SctB), which are highly conserved among *Yersinia* species (Ivanov et al. 2008). Both active and passive immunisation with anti-YopB/YopD serum conferred protection against lethal *Y. pestis* challenge in mice.

#### Promises and challenges associated with targeted inhibition of T3SS

Targeting the T3SS in *Yersinia* offers a compelling anti-infective strategy, with several promising avenues. Small molecules such as SAHs and SABs have been identified by HTS that inhibit T3SS assembly or effector translocation without affecting bacterial viability. Structure-guided approaches have yielded inhibitors of YopH and the SctN ATPase, while antibody-based strategies targeting LcrV and translocon proteins (YopB and YopD) have demonstrated protective efficacy in animal models. These approaches should have the capacity to reduce selective pressure for resistance and preserve the host microbiota, aligning with sustainable antimicrobial goals.

However, significant challenges remain. Many HTS hits lack defined molecular targets, complicating optimization and risking off-target effects. Structure-based inhibitors often fail to translate into cellular or *in vivo* efficacy due to poor permeability or metabolic instability. Antibody therapies face hurdles of antigenic variability, as exemplified by LcrV polymorphisms that limit cross-species protection, as well as delivery constraints. Moreover, while T3SS inhibition attenuates virulence, it does not eliminate bacteria, raising concerns about persistence and the potential for chronic infection. Nonetheless, this limitation may be partly offset by the principle that a robust immune system is better equipped to clear pathogens whose T3SS has been chemically disabled, as the host is no longer confronted with the full array of T3SS-mediated immune-evasion mechanisms. Significantly, no T3SS inhibitor has yet advanced to clinical trials, reflecting these translational barriers. As of now, most data are derived from animal models or *in vitro* studies. However, there is hope for the small-molecule inhibitor Fluorothiazinon (FT): inhibition of T3SS in *Salmonella, Chlamydia*, and *Pseudomonas aeruginosa* has been demonstrated *in vitro*, while in murine models FT significantly reduced *Salmonella* infection when administered both prophylactically and therapeutically (Zigangirova et al. 2021).

A further dimension comes from recent insights into how *Yersinia* behaves under prolonged in-host selective pressure. A longitudinal clinical study showed that *Yersinia enterocolitica* can undergo substantial within-host genome remodelling, including regulatory rewiring, reduced genome content, and altered antimicrobial susceptibility, during chronic infection under sustained antibiotic exposure (*Savin* et al. *2025*). While this study did not report loss of the T3SS itself, it demonstrates that *Yersinia* is evolutionarily flexible and capable of adapting in ways that support persistence when pressured by antimicrobial interventions. In this light, concerns arise that chemical inhibition of the T3SS could create selective conditions that favour bacterial states optimized for long-term survival or recurrence rather than clearance.

This concern gains conceptual support from *Pseudomonas aeruginosa*, where naturally occurring T3SS-negative strains belonging to Clade 5 have been identified (*García-Reyes* et al. *2025*). These strains have completely lost their T3SS gene cluster yet remain viable and continue to produce alternative virulence-associated metabolites, including elastase LasB, pyocyanin, rhamnolipids, and pyoverdine. Their existence illustrates how a Gram-negative pathogen can discard a major virulence system entirely when ecological or evolutionary pressures allow, relying instead on other mechanisms to maintain fitness. While *Yersinia* has not been observed to lose the T3SS, the *Pseudomonas* precedent underscores the broader evolutionary principle: when one virulence system is suppressed, whether by antibiotics or targeted inhibitors, pathogens with flexible genomes can shift toward alternative strategies that maintain survival and potentially persistence (*Savin* et al. *2025*).

Adding to this adaptability, bacteria coordinate their virulence systems through intricate signalling networks. Enteropathogenic *Yersinia*, for instance, inversely regulate T3SS activity and motility (Bleves et al. 2002 et al. 2002, Avican et al. et al. 2015), illustrating how antagonistic relationships between virulence modules enable transitions between distinct physiological and ecological lifestyles. Consequently, inhibiting the T3SS may shift bacterial gene expression toward alternative virulence systems that support persistence, interbacterial competition, or niche establishment even when the T3SS apparatus is blocked. More broadly, overlapping and hierarchically organised regulatory circuits likely permit functional compensation whenever a single virulence mechanism is impaired. Such redundancy underscores a central principle in Gram-negative pathogens: targeting one secretion system may reshape the virulence repertoire rather than suppress it outright, potentially reducing clearance without preventing adaptive reprogramming.

These considerations emphasize that while T3SS inhibition in *Yersinia* remains mechanistically attractive, the evolutionary flexibility of pathogens warns against reliance on single-target anti-virulence strategies. Combination regimens that pair T3SS inhibitors with inhibitors of other virulence systems, conventional antibiotics, or immune modulators may enhance efficacy and reduce the likelihood of compensatory virulence shifts. Lessons from drug-development efforts against other secretion systems also support this multidimensional approach, including type II secretion system inhibitors (Massai et al. 2019) and type IV secretion system inhibitors with dual activity against bacterial conjugation and resistance-gene transfer (Boudaher and Shaffer 2019). Ultimately, translating T3SS-targeted therapies into clinically meaningful interventions will require coordinated development of complementary inhibitors, integrated screening strategies, robust *in vivo* validation, and pharmacological optimisation, ensuring that secretion-system inhibition functions not as a stand-alone intervention but as a component of a broader, multi-pronged anti-infective strategy.

### The CpxA-CpxR two-component regulatory system as a lifestyle switch

The CpxA-CpxR two-component regulatory system (TCRS) is a conserved envelope stress response mechanism in Gram-negative bacteria, comprising the sensor kinase CpxA and the response regulator CpxR (Raivio 2014, Cho et al. 2023, Wan et al. 2024). Upon detecting stress signals such as misfolded periplasmic proteins or membrane perturbations, CpxA autophosphorylates and donates the phosphate to CpxR, which then modulates transcription of genes involved in protein folding, degradation, and virulence regulation. In addition, the Cpx-signalling system modulates bacterial fitness and virulence across diverse pathogens. In *Escherichia coli*, Debnath and colleagues demonstrated that Cpx-signalling is essential for the virulence of Uropathogenic strains UTI89 and CFT073 (Debnath et al. 2013). Its deletion impaired UTI89 bladder colonisation in mice and reduced CFT073 virulence in zebrafish embryos, correlating with diminished host cell invasion and increased susceptibility to complement-mediated killing and amikacin. In *Salmonella enterica*, Cpx-signalling represses expression of the SPI-2 pathogenicity island by directly regulating the SsrAB system (Leon-Montes et al. 2022). It also destabilises the SPI-1 pathogenicity island regulator HilD, thereby downregulating both SPI-1 and SPI-2 under SPI-1-inducing conditions (Fabrega and Vila 2013), underscoring its role in intracellular survival. In *Vibrio cholerae*, Cpx activation downregulates ToxT and TcpP to suppress major virulence factors such as cholera toxin and toxin-coregulated pilus. This repression is largely abolished in a cyclic AMP receptor protein-deficient mutant (Acosta et al. 2015). In *Y. pseudotuberculosis*, Cpx-signalling inhibits biofilm formation by repressing the *hmsHFRS* locus and its regulators HmsC, HmsT, and HmsP, thereby reducing production of Hms-EPS, an exopolysaccharide (EPS) essential for adhesion on both abiotic and biotic surfaces (Gahlot et al. Gahlot, 2022b). Importantly, Hms-EPS corresponds to poly-β-1,6-N-acetyl-D-glucosamine (PNAG) (*Bobrov* et al. *2008*, *Erickson* et al. *2008*), a widely conserved EPS present in many Gram-negative and Gram-positive bacteria, where it functions as a major structural component of biofilms and contributes to environmental persistence and immune evasion. Cpx-signalling also activates RovM, a global regulator of biofilm and virulence (Thanikkal et al. 2019), while repressing RovA (Liu et al. 2011), leading to impaired host cell contact (Carlsson et al. Carlsson, 2007a), and downregulation of the Ysc-Yop T3SS (Carlsson et al. 2007b, Liu et al. 2012). Collectively, these findings highlight the multifaceted role of Cpx-signalling in regulating virulence, EPS-mediated biofilm biology, and stress responses, positioning it as a promising target for antimicrobial intervention strategies.

#### Cpx-signalling as a target for antimicrobial development

The Cpx envelope stress response system plays a critical role in the virulence and antibiotic resistance of *Y. pseudotuberculosis*, regulating outer membrane integrity and lipid A remodelling. These functions contribute to resistance against last-resort antibiotics such as colistin and polymyxin B (Gahlot et al. 2024). Given its conservation across Enterobacteriaceae, the Cpx system represents a promising target for antimicrobial development. Beyond *Yersinia*, the Cpx envelope stress response system is conserved across Enterobacteriaceae and is present in several drug-resistant pathogens, including *E. coli, Klebsiella pneumoniae, Neisseria gonorrhoeae*, and *Haemophilus ducreyi* (Srinivasan et al. 2012, Bontemps-Gallo et al. 2015, Dbeibo et al. 2018). Components of this system have emerged as potential targets for antimicrobial development, particularly by inhibiting CpxA phosphatase activity or activating the CpxR regulator. Disrupting these elements compromises bacterial stress responses, enhancing susceptibility to host immunity and antibiotics.

Targeting CpxA in *E. coli* reduces virulence and increases antibiotic sensitivity, highlighting its therapeutic potential (van Rensburg et al. 2015, Dbeibo et al. 2018, Fortney et al. 2022). Activation of CpxR in phosphatase-deficient *cpxA* mutant backgrounds suppresses virulence factor expression in multiple pathogens, consistent with its role in regulating periplasmic protein traffic (Raivio 2014, Cho et al. 2023, Wan et al. 2024). In *H. ducreyi, cpxA* mutants downregulate seven key virulence determinants and are avirulent in human skin, while *cpxR* mutants retain full virulence (Spinola et al. 2010, Labandeira-Rey et al. 2011). Similarly, constitutive Cpx activation in *S. enterica* serovar Typhimurium abolishes virulence in mice. Wild-type and *cpxR* mutants cause infection, whereas phosphatase-deficient *cpxA* mutants do not (Humphreys et al. 2004). Moreover, in murine models of Uropathogenic *E. coli* and *N. gonorrhoeae, cpxA* mutants are outcompeted by wild-type strains by three orders of magnitude (Debnath et al. 2013, Gangaiah et al. 2017).

These findings support Cpx activation as a broad-spectrum anti-virulence strategy. A HTS study of 36 000 compounds identified a nitro-containing molecule that activates CpxR∼P-dependent transcription in *E. coli*, likely by inhibiting CpxA phosphatase activity (van Rensburg et al. 2015). Structural modification of this compound yielded a serum-stable, less cytotoxic variant. Such screens can be expanded to identify additional Cpx activators or modulators of other two-component systems.

#### Challenges and future directions in targeting TCRS

Targeting the Cpx-signalling presents a promising strategy for developing novel antimicrobial agents. However, several challenges must be addressed to translate this approach into effective clinical therapies. One major concern is the potential for off-target effects, as the Cpx system regulates a broad range of cellular processes beyond stress response and virulence, including envelope homeostasis and protein folding. Inhibiting this pathway indiscriminately could disrupt essential bacterial functions or affect commensal microbiota. Additionally, the risk of resistance development to Cpx-targeted inhibitors may compromise their long-term efficacy.

To overcome these limitations, further research is needed to elucidate the precise molecular mechanisms of CpxA-CpxR signalling and its role in bacterial pathogenesis. This knowledge will support the design of more selective inhibitors with minimal off-target activity. Moreover, combination therapies targeting multiple regulatory pathways or pairing Cpx inhibitors with conventional antibiotics may enhance antimicrobial efficacy and reduce the likelihood of resistance emergence. *Yersinia* species, particularly *Y. enterocolitica* and *Y. pseudotuberculosis*, serve as effective model organisms for studying the molecular mechanisms of the CpxA-CpxR TCRS. These pathogens possess a well-characterised Cpx system that is highly conserved among many Gram-negative bacteria, making them ideal for dissecting regulatory dynamics. Additionally, *Yersinia* species are genetically tractable and exhibit robust stress responses under laboratory conditions, allowing for detailed mechanistic studies.

Research on *Yersinia* can significantly enhance our understanding of bacterial stress adaptation, especially in the context of host-pathogen interactions. Insights gained from well-characterised *Yersinia* infection models can inform the development of targeted therapies that disrupt stress response pathways, attenuate virulence, and sensitise bacteria to existing antibiotics (Fig. 1). Furthermore, studying the Cpx system in *Yersinia* may reveal conserved regulatory motifs and potential drug targets applicable across a broad spectrum of pathogenic bacteria.

In conclusion, while challenges remain, targeting the Cpx-signalling pathway offers a compelling avenue for antimicrobial development. By disrupting bacterial stress adaptation and attenuating virulence, Cpx modulators could potentiate existing antibiotics and provide new tools against MDR pathogens. Continued investigation into the biology of TCRS, particularly through model organisms like *Yersinia*, will be critical for advancing this strategy toward clinical application.

### Multifactorial anti-infective strategies are possible

Pathogenic *Yersinia* spp. employs a diverse arsenal of virulence factors to colonise hosts, evade immune responses, and cause disease. While the T3SS has long been the focal point of virulence research and therapeutic targeting, recent advances underscore the importance of alternative systems that contribute to pathogenesis. These include quorum sensing (QS), adhesive structures and their assembly pathways, metabolic adaptation, solute transport, and additional secretion systems. Targeting these pathways offers a promising anti-infective strategy that may reduce selective pressure for resistance and preserve the host microbiota.

#### Quorum sensing: coordinating virulence at the population level

Exploring QS inhibition holds promise for the development of anti-infective compounds to effectively treat *Yersinia* and other bacterial infections. QS is a cell-to-cell communication system employed by bacteria to regulate gene expression based on population density. In *Yersinia* spp., particularly *Y. pseudotuberculosis and Y. enterocolitica*, prominent QS systems are the LuxIR-type that modulate biofilm formation, motility, and environmental persistence (Atkinson et al. 2006a). Key quorum-sensing autoinducers are the acyl-homoserine lactones (AHLs) and include N-(3-oxohexanoyl)-homoserine lactone (3-oxo-C6-HSL), N-hexanoyl-HSL (C6-HSL), and N-octanoyl-HSL (C8-HSL), along with additional longer-chain 3-oxo-substituted AHLs such as 3-oxo-C7-HSL and 3-oxo-C8-HSL identified via LC–MS profiling (*Ortori* et al. *2007*). These AHLs are synthesized and perceived through conserved LuxIR-type homologues, including the paired YpsI/YpsR and YtbI/YtbR systems in *Y. pseudotuberculosis* (*Atkinson* et al. *1999*) and the YenI/YenR system in *Y. enterocolitica* (Atkinson et al. 2006b), which collectively regulate motility, clumping, and multiple QS-dependent traits.

Inhibiting such systems can disrupt prominent communication networks, impeding the coordinated expression of virulence genes. For example, targeting the AHL autoinducer molecules can interfere with the QS system in *Yersinia*, hindering the synchronised release of virulence factors. This disruption compromises the bacteria’s ability to establish infections and evade host defences. Several studies have demonstrated the efficacy of QS inhibitors (QSIs) in attenuating bacterial virulence. Compounds like furanones and synthetic analogues have shown promise in mitigating the pathogenicity of various bacterial species by interfering with QS systems (Rasmussen et al. 2005). Recent research has expanded the understanding of QS-regulated virulence in other Gram-negative pathogens and the potential of QSIs as broad-spectrum anti-infective agents. For instance, natural and synthetic QSIs have been shown to inhibit AHL-mediated signalling in multiple species, including *P. aeruginosa* and *Vibrio* spp., with implications for cross-species applications (Naga et al. 2023). Moreover, structure-activity relationship studies have identified key chemical scaffolds that effectively block QS-regulated phenotypes such as biofilm formation and toxin production (Hetta et al. 2024). In *Yersinia*, although QS is less studied than in other pathogens, the conservation of AHL-based systems suggests that these inhibitors may be repurposed or optimised for use in this genus (Sivasankar et al. 2020). Utilising QS inhibition offers advantages for managing *Yersinia* infections, including the potential for developing broad-spectrum anti-infective compounds and minimizing the risk of resistance, though specificity and off-target effects on beneficial microbiota remain challenges.

#### Adhesins, fimbriae, and the chaperone–usher assembly pathway

Bacteria produce a diverse array of adhesins, including pili and fimbriae, which can be classified based on their assembly pathways. These include chaperone-usher (CU) pili, type V pili, type IV pili, curli and Fap fibers, conjugative and type IV secretion pili, as well as sortase-mediated pili (Proft and Baker 2009, Lukaszczyk et al. 2019, Gahlot et al. 2022a). These structures fulfil multiple roles in bacterial physiology, contributing to adhesion, host cell invasion, DNA and protein secretion and uptake, biofilm formation, and motility.

*Yersinia* species also express several adhesive organelles that mediate interactions with extracellular matrix components and host cells (Mikula et al. 2013, Chauhan et al. 2016). These organelles are essential for host tissue colonisation and are often regulated in response to environmental stimuli. Due to their surface exposure and immunogenic properties, they represent promising targets for vaccine development and antibody-based therapies. However, functional redundancy among adhesins may necessitate combinatorial approaches to achieve effective inhibition.

Among the major assembly pathways, the CU system is particularly well-characterised (Waksman and Hultgren 2009, Busch and Waksman 2012). The CU pathway involves periplasmic chaperones that stabilise pilus subunits and usher proteins that mediate their assembly and translocation across the outer membrane. In *Yersinia*, this includes the pH6 antigen (PsaA), which is critical for adhesion and immune evasion (Pakharukova et al. 2016). Another well-studied CU system is the antiphagocytic F1 capsular antigen of *Y. pestis*. The Caf1-based capsule is a major virulence factor. Caf1R-regulated expression at 37 °C enables assembly through the Caf1M (chaperone) and Caf1A (usher). F1 fibres block phagocytosis and enhance survival, with folding energy driving stable, immunogenic structures essential for pathogenesis and vaccine design (Zavialov et al. 2003, Runco et al. 2008, Gahlot et al. 2021) (see also section concerning *Yersinia* vaccine development).

Foundational studies in *E. coli* elucidated the molecular mechanisms of CU-mediated pilus assembly, particularly the interactions between chaperones and subunits (Waksman and Hultgren 2009, Busch and Waksman 2012). These insights have facilitated the development of small-molecule inhibitors that disrupt these interactions, thereby preventing pilus assembly without affecting bacterial viability (Psonis and Thanassi 2019, Sarshar et al. 2020). Such compounds have shown efficacy in reducing adhesion, biofilm formation, and virulence *in vivo*. Given the structural conservation of the CU pathway across Enterobacteriaceae, targeting CU pili represents a rational, resistance-sparing anti-infective strategy with strong translational potential in *Yersinia*.

#### Targeting sessile *Yersinia* communities

Biofilm biology plays fundamentally different roles across the *Yersinia* genus, and these differences dictate whether sessile communities are relevant targets for therapeutic or ecological intervention (Fig. 1). In *Yersinia pestis*, biofilm formation is tightly linked to its unique flea-borne transmission strategy (*Hinnebusch* et al. *2021*). Within the flea foregut, *Y. pestis* produces a dense, Hms-dependent extracellular polysaccharide matrix that blocks the proventriculus, triggering a starvation response that drives repeated feeding attempts and enhances transmission to rodent hosts. This biofilm phenotype is governed by a dedicated regulatory network, including the *hmsHFRS* operon and c-di-GMP signalling enzymes such as HmsT, HmsD, and HmsP, with additional contributions from post-transcriptional regulators like the sRNA HmsB, which promotes exopolysaccharide production and matrix stabilization (Liu and Zheng 2019). Recent genomic analyses further reveal that environmental selection pressures influence biofilm capacity; for example, allelic variation in the RNA polymerase ω-subunit gene *rpoZ* modulates biofilm formation in response to ecological conditions such as colder, drier climates, highlighting the adaptive nature of this trait within vector habitats (Cui et al. 2020).

Despite its ecological importance, *Y. pestis* biofilm formation is not expressed during mammalian infection, where the bacterium adopts a planktonic extracellular lifestyle. Because biofilms are essentially absent in the mammalian host, they do not constitute a viable therapeutic target in clinical plague management. Any attempt to disrupt *Y. pestis* biofilm formation would apply not to treating human disease but to interrupting transmission in the flea, which constitutes an ecological rather than medical intervention.

In contrast, biofilm formation in the enteropathogenic species *Yersinia enterocolitica* and *Y. pseudotuberculosis* plays a major role in their ability to persist and circulate as free-living bacteria in the environment (Naktin and Beavis 1999, Tauxe 2004, *Shoaib* et al. *2019*). *Y. enterocolitica* readily forms biofilms on abiotic surfaces such as silicone and plastic, and this behaviour is widespread among clinical isolates. In a survey of sixty strains, all could produce biofilms, and these sessile communities displayed markedly increased tolerance to antimicrobial agents, underscoring the protective and persistent nature of the biofilm state in environmental contexts (*Ioannidis* et al. *2014*). Biofilm formation is also modulated by environmental conditions: at 26 °C, which is the temperature relevant to soil, water, and food-associated habitats, *Y. enterocolitica* enhances biofilm production, initially through motility-driven surface attachment followed by robust extracellular polymer synthesis (Oh and Kim 2025). These processes reveal a regulatory system that allows the bacterium to alternate between motility-dominated and matrix-dominated biofilm strategies depending on its surroundings. These properties allow enteropathogenic *Yersinia* to establish long-lived environmental reservoirs in water systems, soil, and food-processing environments (Oh and Kim 2025). Such reservoirs can serve as persistent contamination sources capable of initiating new infections, particularly via foodborne and waterborne routes. From an ecological standpoint, these biofilm-associated reservoirs form an essential component of the transmission landscape, supporting the continued circulation of enteropathogenic *Yersinia* outside animal hosts. While these reservoirs are not amenable to direct therapeutic targeting within infected individuals, their management is highly relevant to public health and environmental control. In this context, it is worth noting that bacteriophage-based approaches have been explored as potential environmental biocontrol strategies against *Yersinia* species. These applications, which belong to the realm of ecological management rather than clinical therapy, are discussed in greater detail in the section *Yersinia Phage Therapy*.

In summary, biofilm formation in *Y. pestis* represents a vector-specific adaptation essential for flea-borne transmission but irrelevant to mammalian infection, while in the enteropathogenic species, biofilms promote environmental persistence and support reservoirs capable of seeding new infections. Although these sessile communities are central to the ecology of *Yersinia*, their suitability as intervention points depends heavily on whether the focus is clinical treatment, environmental sanitation, or vector-borne transmission control.

#### Alternative protein secretion systems as drug targets

In addition to the well-characterised T3SS, *Yersinia* spp. utilises several other secretion systems to deliver virulence factors and adapt to host environments. Type 1 (T1SS), Type 2 (T2SS) and Type 5 (T5SS) secretion transports a wide range of proteins, including toxins, enzymes, and adhesins. Type 6 secretion system (T6SS) contributes to interbacterial competition and may modulate host interactions. Finally, type 4 secretion system (T4SS), although less studied in *Yersinia*, is a known virulence determinant in other Gram-negative pathogens and plays a key role in horizontal gene transfer. Recent work by Massai and colleagues demonstrated the feasibility of HTS for inhibitors of the T2SS in *P. aeruginosa*, identifying compounds that impair secretion without affecting bacterial growth (Massai et al. 2019). These findings provide a valuable framework for developing anti-infective agents targeting conserved secretion systems in *Yersinia*. Moreover, T4SS inhibitors have garnered attention not only for their ability to block virulence factor translocation but also for their potential to inhibit bacterial conjugation and the spread of antibiotic resistance genes. Dual-purpose inhibitors that target both virulence and horizontal gene transfer represent a promising strategy in the fight against multidrug-resistant pathogens (Boudaher and Shaffer 2019). Taken together, the pronounced structural conservation within secretion systems of the same type highlights their value as therapeutic targets, and the translational potential of inhibitors directed against them warrants further investigation.

#### Y. pseudotuberculosis as a model for *in vivo* metabolic adaptation

Leading research from Umeå University, Sweden, positions *Y. pseudotuberculosis* as a powerful model organism for dissecting bacterial metabolic adaptation during host infection. Advanced *in vivo* transcriptomic analyses were employed to characterise gene expression patterns of *Y. pseudotuberculosis* within host tissues, revealing a dynamic reprogramming of bacterial metabolism in response to environmental stressors and immune pressure (Avican et al. 2015). One of the key findings stemming from this study is that during persistent infection, *Y. pseudotuberculosis* downregulates classical virulence genes while upregulating genes involved in stress resistance, nutrient acquisition, and metabolic flexibility. This shift supports long-term survival in host niches and reflects a broader strategy of metabolic adaptation that is critical for chronic infection. The study was made possible because the authors developed innovative methodologies for RNA extraction from complex tissue samples, enabling high-resolution analysis of bacterial gene expression *in vivo* (Avican et al. 2021). These approaches have been instrumental in identifying heterogeneous gene expression patterns and regulatory networks that govern metabolic adaptation. The launch of the PATHOgenex database further extends this work, providing a comparative platform for analysing stress responses across more than 30 human pathogens (Fernandez et al. 2024).

Importantly, the metabolic pathways identified in *Yersinia* mirror those observed in other clinically relevant pathogens. The pathogen-specific nature of metabolic adaptations presents a unique opportunity for targeted drug development. Unlike broad-spectrum antibiotics, metabolic inhibitors can be designed to selectively disrupt infection-specific pathways, minimising collateral damage to the host microbiota and reducing the likelihood of resistance. Already, recent studies demonstrate promising metabolic targets for drug development for the treatment of bacterial infections, including metabolic enzymes such as pyruvate dehydrogenase (Birkenstock et al. 2012), iron acquisition and storage systems such as ferrosome biosynthesis (Stelitano et al. 2023), lipid biosynthesis and remodelling enzymes (Carter et al. 2025), solute transporters and the acquisition of essential nutrients (Akhtar and Turner 2022) and amino acid biosynthesis and carbon metabolism pathways (Tong and Brown 2023). Inhibiting these pathways can attenuate virulence without affecting bacterial viability *in vitro*, though specificity is crucial to avoid collateral effects on beneficial microbiota and human cells. Hence, these approaches offer a subtle yet effective anti-infective strategy that may synergise with host-directed therapies. Better understanding of bacterial metabolism in the host and its association with virulence (Glass et al. 2024, Bhagwat et al. 2025, Peng et al. 2025), coupled with the advancement of HTS technologies and access to AI and *in silico* screens (Alkatheri et al. 2023, Ayon 2023, Lluka and Stokes 2023, Manshadi et al. 2024, Mudgal et al. 2025, Mulat et al. 2025), will invariably increase the feasibility of targeting niche specific bacterial metabolic signatures as a strategy for developing new antibacterial drugs. This reinforces the utility of *Y. pseudotuberculosis* as an *in vivo* model system for identifying conserved metabolic vulnerabilities that can be targeted across multiple bacterial species.

### Development of host-directed therapies for *Yersinia* infection

Pathometabolism is the term given to the metabolic interplay between intracellular pathogens and host cells (Eisenreich et al. 2015). It offers a strategic framework for the development of host-directed therapies (HDTs) (Fig. 1). *Yersinia* species, particularly *Y. pestis, Y. pseudotuberculosis*, and *Y. enterocolitica*, serve as robust models for investigating host-pathogen metabolic interactions. *Yersinia* is a well-established model to study pathometabolism due to its defined virulence mechanisms, capacity for persistence within the well-established host infection models, and suitability for *in vivo* transcriptomic and proteomic studies (Avican et al. 2015, Nuss et al. 2017).

#### Host metabolic responses to *Yersinia* infection—a foundation for HDT design

Host cells respond to *Yersinia* invasion by reprogramming their metabolism to restrict pathogen access to nutrients and coordinate antimicrobial functions. This includes modulation of glycolysis, mitochondrial activity, and lipid metabolism, which influence cytokine production, phagocytosis, and antigen presentation. Recent omics-based studies have further demonstrated that *Yersinia* infection triggers extensive reprogramming of host metabolic pathways. For example, *Y. pseudotuberculosis* infection alters host cell metabolism, including energy production, lipid biosynthesis, and immune signalling (Nuss et al. 2017). Complementary studies have revealed changes in mitochondrial function and redox balance (Alugubelly et al. 2016), and metabolic shifts in epithelial cells that affect immune responses (Ahlawat et al. 2021). These findings underscore the central role of host metabolism in shaping immune responsiveness to *Yersinia*.

However, the use of cancer-derived cell lines in many studies limits physiological relevance due to deregulated carbon metabolism. To overcome these limitations, more physiologically relevant models are increasingly employed. Primary human macrophages have been used to demonstrate that *Y. enterocolitica* effectors, such as YopP, remodel host epigenetic landscapes, altering histone modifications and transcriptional programs involved in immune signalling and metabolism (Bekere et al. 2021). These models also reveal species-specific immune responses, such as RIPK1-independent apoptosis in human macrophages, which contrasts with murine systems and underscores the importance of human-specific platforms (Nataraj et al. 2024). Intestinal organoids also offer a powerful system for studying gut-associated *Yersinia* species. A recent innovation uses *Yersinia* invasin as a defined, animal-free extracellular matrix to support the long-term culture of primary intestinal epithelial cells. Invasin is a *Yersinia* surface protein that normally promotes bacterial entry by binding tightly to host β1-integrins. In this context, its integrin-binding ability is repurposed as a defined, animal-free extracellular matrix that supports long-term growth and differentiation of primary intestinal epithelial cells, enabling organoid expansion and high-throughput studies of host–pathogen interactions at the mucosal interface (Wijnakker et al. 2025). These and other advanced models enable more accurate mapping of host-pathogen metabolic interactions and immune modulation, thereby enhancing the translational relevance of findings and supporting the development of precision HDTs (Fig. 1).

Another consideration is the central role played by diet and the gut microbiota in shaping host immunity and inflammation (Rooks and Garrett 2016, Graham and Xavier 2023, Arifuzzaman et al. 2024). Consequently, the development of potential HDTs that modulate microbiota-derived metabolites are gaining traction. For example, short-chain fatty acids, such as butyrate produced by microbial fermentation, exhibit anti-inflammatory effects and support gut barrier integrity, while microbial bile acid metabolites interact with host nuclear receptors, influencing cholesterol and steroid metabolism. Moreover, technological advancements enabling deeper insights into individual host–microbiome profiles have propelled the emergence of precision microbiome medicine. This approach seeks to personalise interventions, such as probiotics, prebiotics, symbiotics, and engineered microbes, according to the specific needs of the individual (Daisley and Allen-Vercoe 2024, Ratiner et al. 2024, Shahid 2025). These approaches could be adapted to *Yersinia* infections, particularly in the gut-associated species *Y. enterocolitica* and *Y. pseudotuberculosis*, by enhancing mucosal immunity and limiting pathogen colonisation, or with respect to *Y. ruckeri* infections of fish (see section *Probiotics as an alternative for* Y. ruckeri *infection*).

Another additional factor that may serve as a basis for HDT design is nutritional immunity, which refers to the host’s strategy of sequestering essential nutrients, especially trace metals like iron, zinc, and manganese, to inhibit bacterial growth (Antelo et al. 2021, Murdoch and Skaar 2022, Ullah and Lang 2023). During *Yersinia* infections, the host restricts iron availability through proteins such as ferritin, transferrin, lactoferrin, and hepcidin, which limit both iron uptake and export, contrasting to individuals with iron overload, who are more susceptible to *Yersinia* infection (Champion et al. 2011, Drakesmith and Zoller 2024). Further, NRAMP1 restricts intracellular pathogens, including *Yersinia*, by depleting iron and other essential divalent metal ions within macrophages; as a phagosomal membrane transporter, it exports these ions out of the phagosome, creating a metal-limited phagolysosomal environment that suppresses microbial replication. Although enhancing metal-export mechanisms such as NRAMP1 represents a potential therapeutic strategy, such approaches are complicated by the central role of metal transporters in maintaining systemic metal homeostasis, which is essential for overall human health and extends far beyond infectious disease contexts (Wessling-Resnick 2015). Conversely, because many bacteria depend heavily on iron acquisition for survival, their high-affinity iron uptake systems can be exploited for therapeutic purposes; in particular, siderophore-mediated transport pathways provide an opportunity for targeted delivery of siderophore-conjugated antibiotics, offering an alternative antibacterial strategy (Rayner et al. 2023, Moro 2025). These strategies are particularly relevant for *Yersinia*, which relies on high-affinity iron acquisition systems for virulence and survival (Perry and Fetherston 2011, Rakin et al. 2012).

#### Immune-modulating HDTs

Modulation of host immunity in response to infection is a promising HDT strategy for severe bacterial infections, including systemic *Yersinia* disease, where immune evasion and dysregulated inflammation contribute to pathogenesis. HDTs aim to restore immune homeostasis and enhance antimicrobial functions without relying solely on pathogen-targeted antibiotics, which is critical given the rising threat of MDR (Taya et al. 2023, Cai et al. 2025, Luo et al. 2025).

Mechanistically, several approaches have been explored. Enhancing phagolysosome maturation and autophagy can improve intracellular clearance of pathogens. *Y. pestis* and *Y. enterocolitica* actively subvert autophagy by preventing vacuole acidification and lysosomal fusion, creating niches for replication (Deuretzbacher et al. 2009, Alem et al. 2015). Pharmacologic activation of autophagy or inhibition of the Ysc-Yop T3SS effectors could reverse this blockade. Similarly, immune checkpoint blockade (e.g. PD-1/PD-L1, CTLA-4 inhibitors) has shown potential to reinvigorate exhausted T cells in chronic infections, though its application in bacterial disease remains experimental; preclinical data suggest that restoring T-cell effector function could enhance clearance of persistent pathogens (Henriksen et al. 2025).

Macrophage polarisation toward an M1 phenotype is another HDT target. Activated macrophages exhibit enhanced nitric oxide production and cytokine secretion, shifting *Yersinia*-induced apoptosis toward caspase-1-dependent pyroptosis, which promotes pathogen clearance and IL-18 release (Bergsbaken and Cookson 2007). Conversely, *Yersinia* employs T3SS effectors (YopP/J, YopM) to suppress NF-κB signalling, inflammasome activation, and cytokine production, thereby dampening macrophage-mediated immunity (Schubert et al. 2020, Bliska et al. 2021). Hence, HDTs targeting pathways that boost NF-κB or inhibit Yop-mediated kinase hijacking could restore macrophage antimicrobial activity. Regulation of cytokine networks is critical to prevent immunopathology. Recent work highlights a TNF–IL-1β axis within intestinal pyogranulomas that restricts *Yersinia* growth and disruption of this circuit leads to uncontrolled infection (Matsuda et al. 2024). This means that HDTs enhancing TNFR1 or IL-1 signalling could improve outcomes. Similarly, modulation of TLR4–TRIF signalling influences macrophage apoptosis and inflammation during *Yersinia* infection (Peterson et al. 2016, Yow et al. 2024), offering another potential therapeutic node. Collectively, the strategies of autophagy induction, checkpoint inhibition, macrophage reprogramming, cytokine modulation, and inflammasome regulation represent rational HDT approaches for severe *Yersinia* infections.

#### Host receptors as entry points and therapeutic targets

A complementary approach to modulating host metabolism and enhancing innate and adaptive immune responses involves targeting host cell-surface receptors that mediate pathogen entry. These receptors not only determine tissue and cell tropism but also influence susceptibility and disease severity. Host cell surface receptors play a critical role in *Yersinia* pathogenesis and represent promising targets for HDTs. While *Y. pestis* has lost several adhesins, such as invasin and YadA, it retains the LcrV protein, which binds to the formyl peptide receptor 1 (FPR1) on immune cells, facilitating effector delivery via the T3SS (Osei-Owusu et al. 2019). Specifically, FPR1 is a G-protein–coupled innate immune receptor on neutrophils and other myeloid cells that detects N-formylated peptides from bacteria or damaged mitochondria, triggering rapid chemotaxis and antimicrobial activation, including ROS production and inflammatory signalling. FPR1 functions as a host receptor for *Yersinia pestis*, and genetic variation in this receptor influences host susceptibility to plague; for example, the FPR1^R190W^ allele provides partial resistance by reducing neutrophil targeting and effector translocation by the *Y. pestis* type III secretion system (Osei-Owusu et al. 2019). In parallel, a cellular genome wide association study identified a nonsynonymous FCRL3 variant, rs2282284 (N721S), which enhances bacterial attachment and invasion of B cells by altering a tyrosine-based signalling motif (Keener et al. 2025). Together, these findings highlight that host genetic variation across multiple immune receptors, FPR1 on phagocytes and FCRL3 on lymphocytes, can modulate both susceptibility and cell-type specificity of *Y. pestis* infection.

Using host receptors to establish specific infection niches is a strategy shared across many bacterial pathogens. In *E. coli*, allelic variation in the *fimH* adhesin consists primarily of nonsynonymous point mutations that cluster in structural “hot-spot” positions and alter the mannose-binding pocket, thereby shifting binding affinity and shear-dependent adhesion properties (*Sokurenko* et al. *2004*). These mutations produce pathoadaptive variants that enhance colonization of alternative host tissues, most notably the uroepithelium, while often reducing fitness in the intestinal niche, resulting in clear niche-associated patterns of tissue tropism. A similar principle applies to *Salmonella enterica*, where point mutations in *fimH* likewise modulate epithelial binding and are associated with increased systemic invasiveness, indicating that FimH-mediated ligand-binding shifts also drive niche expansion in this genus (Kisiela et al. 2012). *Listeria monocytogenes* uses Internalin A to bind E-cadherin in a species-specific manner, demonstrating yet another example of receptor-targeted specificity (Lecuit et al. 1999). *Staphylococcus aureus* adapts to rabbit hosts through mutations in *dltB* that alter cell-wall composition and antimicrobial peptide resistance (Malachowa et al. 2022), and many *S. aureus* strains also carry phage-derived immune modulators influencing host interactions (Ojiogu et al. 2025). Collectively, these systems show that pathogens repeatedly evolve mutations in their adhesins to exploit specific host receptors, thereby driving tissue tropism and niche specialization. This evolutionary pattern underscores a critical therapeutic insight: although bacteria diversify their ligands, the host receptors they converge upon remain comparatively stable and thus represent the more strategic intervention points. In this context, receptors targeted by *Yersinia*, including FPR1 and FCRL3, are especially compelling candidates for host-directed therapies, because modulating the host receptor can block pathogen entry regardless of the specific bacterial mutations that arise.

#### Future directions in host-directed therapies

While no HDTs currently exist that specifically target host metabolism in the context of *Yersinia*, the mechanistic insights gained from omics-based studies suggest several avenues for therapeutic exploration (Fig. 1). Future research should focus on identifying key metabolic nodes that regulate immune function during *Yersinia* infection, validating these targets in physiologically relevant *in vivo* models, and assessing the therapeutic potential of metabolic modulators, perhaps in combination with conventional antibiotics. Integration of multi-omics data with functional immunometabolic assays will be essential for translating these insights into clinically actionable interventions. Furthermore, as personalised medicine gains traction, knowledge of individual genetic predispositions and immune responses to infection may eventually guide targeted interventions that improve outcomes. By acting on conserved host pathways, HDTs offer cross-species efficacy and resilience against antimicrobial resistance. Continued research into host-pathogen-microbiota metabolic interactions and the use of physiologically relevant cellular models will be essential for advancing precision therapies.

## *Yersinia* phage therapy

Phage therapy exploits the ability of bacteriophages to selectively infect and lyse bacterial cells, providing an alternative or complement to antibiotics, particularly in the context of rising MDR and biosecurity concerns (Leon-Velarde et al. 2019, Goladze et al. 2024, Guo et al. 2024). Importantly, therapeutic applications rely exclusively on strictly lytic (virulent) phages, as temperate phages can integrate into bacterial genomes, transfer virulence or antimicrobial-resistance genes, and therefore are unsuitable and unsafe for clinical use. Individual phages exhibit distinct host specificity, enabling targeted interventions. Recent studies have demonstrated encouraging results for phage therapy against *Yersinia* infections. Experimental work with pneumonic plague models has shown that a four-phage cocktail (YPP-401) can provide substantial post-exposure protection, even when administered up to 18 hours after infection onset, highlighting its potential as an emergency countermeasure (Vagima et al. 2022, Kilgore et al. 2024). Effective phage therapy against *Y. enterocolitica* represents a promising approach for food safety, reducing contamination and preventing human yersiniosis in the processing industry (Hammerl et al. 2024). Moreover, phages targeting *Y. ruckeri* are relevant for aquaculture, where they could reduce antibiotic use and limit disease outbreaks in fish farming (Welch 2020). These findings underscore the feasibility of phage therapy across clinical, veterinary, and food processing settings, provided that delivery systems and stability under physiological or aquatic conditions are optimised (Ryan et al. 2011, Hesse and Adhya 2019, Palma and Qi 2024) (Fig. 1).

However, the narrow host range of phages and the rapid emergence of resistance represent significant limitations to their therapeutic application. Phages typically exhibit high specificity, often infecting only certain strains within a species, which complicates the treatment of genetically diverse bacterial populations (Pires et al. 2020, Holtappels et al. 2023). This specificity, while advantageous for preserving the microbiota, means that a single phage or even a small cocktail may fail if the infecting strain lacks the required receptor or mutates to avoid adsorption. Resistance can arise within hours to days through receptor modification, loss of receptors, or acquisition of anti-phage defence systems such as CRISPR-Cas (Andrews and Fields 2021, Bleriot et al. 2024). In *Yersinia*, resistance has been linked to mutations in lipopolysaccharide biosynthesis genes, which can serve as phage receptors (Filippov et al. 2011, Xiao et al. 2023). While such mutations often impose fitness costs or attenuate virulence, reducing pathogenic potential, they still pose a challenge for sustained efficacy. Strategies to overcome these limitations include the use of cocktails targeting multiple receptors, engineering phage with expanded host ranges, and combining phage with antibiotics to exploit synergistic effects (Ryan et al. 2011, Hesse and Adhya 2019, Palma and Qi 2024). Advances in phage engineering and the incorporation of depolymerases to disrupt biofilms further enhance therapeutic potential (Guo et al. 2023).

In summary, the prospects of *Yersinia* phage therapy are shaped by advances across human medicine, food safety, and aquaculture. In clinical settings, phage therapy is poised to function primarily as an adjunct to antibiotics for infections caused by MDR strains and as a biodefense measure against plague (*Vagima* et al. *2022*, *Kilgore* et al. *2024*). In food safety, phages targeting *Y. enterocolitica* offer a means to reduce contamination risks in meat and dairy production (*Hammerl* et al. *2024*). Within aquaculture, phage-based control of *Y. ruckeri* represents a sustainable alternative to antibiotics, improving fish health and minimizing environmental impact (Welch 2020). Broader ecological applications, such as reducing *Yersinia* reservoirs in water and soil, align with One Health strategies but will require rigorous environmental risk assessments. To progress toward widespread implementation, robust regulatory frameworks, large-scale clinical trials, and standardized production and quality-control protocols are essential. Ultimately, continued research into phage–host interactions, resistance evolution, and effective delivery systems will determine how fully phage therapy can be integrated across clinical, veterinary, and industrial settings (*Pires* et al. *2020*, *Holtappels* et al. *2023*).

## *Yersinia* vaccine development

Vaccine development against *Yersinia* spp. remains a critical area of research (Fig. 1), particularly due to the biothreat potential of *Y. pestis*, the etiological agent of plague. While *Y. enterocolitica, Y. pseudotuberculosis*, and *Y. ruckeri* are primarily associated with enteric or aquatic infections, *Y. pestis* poses a unique threat due to its historical lethality and potential for aerosol transmission. Consequently, vaccine strategies against *Y. pestis* have focused on both subunit and live attenuated platforms.

### An updated view on *Y. pestis* vaccinology

Subunit vaccines targeting *Y. pestis* have primarily focused on the F1 capsular antigen and the LcrV virulence protein, both of which elicit robust humoral responses and confer protection in preclinical models (Little et al. 2008, Boyer et al. 2010, Carrillo-Conde et al. 2010, Rosales-Mendoza et al. 2010, Wang et al. 2020, Biryukov et al. 2021). The F1-V fusion protein has demonstrated synergistic immunogenicity and is a leading candidate for plague prophylaxis (Powell et al. 2005, Chichester et al. 2009). On the other hand, live attenuated vaccines offer the advantage of broad and durable immunity but raise safety concerns, particularly regarding reversion to virulence. Attenuated *Y. pestis* strains with targeted deletions in virulence loci (e.g. *pla, caf1, lcrV*) have shown promise in balancing safety and immunogenicity (Garmory et al. 2003, Russmann et al. 2003, Bubeck and Dube 2007, Sun et al. 2011, Wang et al. 2013, Sanapala et al. 2016, Zauberman et al. 2019, Derbise et al. 2020, Feng et al. 2020, Cote et al. 2021). However, regulatory hurdles and biosafety concerns limit their clinical translation (Singh et al. 2019).

Recent advances in *Y. pestis* vaccine development reflect a shift toward integrated, multi-modal strategies that address both immunogenicity and safety. Subunit vaccines targeting conserved virulence factors, particularly the F1 and LcrV antigens of *Y. pestis*, remain the most promising candidates for near-term deployment. The F1-V fusion protein continues to demonstrate robust protection in preclinical models, including against pneumonic plague. Emerging strategies now explore heterologous vaccination, combining live attenuated *Y. pestis* strains with subunit components to enhance immune breadth and durability. For example, Biryukov and colleagues reported that combining attenuated *Y. pestis* mutants (e.g. Δ*yscN* or Δ*pgm*) with rF1V or rV subunits significantly improved protection, even against nonencapsulated strains (Biryukov et al. 2023). In parallel, vector-based platforms have gained traction. A recent study introduced a recombinant *Francisella tularensis* LVS Δ*capB* vector expressing *Y. pestis* F1 and LcrV antigens, which conferred complete protection in murine pneumonic plague models and outperformed the traditional EV76 vaccine in both efficacy and safety (Jia et al. 2025).

Despite these advances, no plague vaccine is currently licensed for public use. Most candidates remain in Phase 2 trials, and development is largely driven by biodefense priorities. Key barriers include the rarity of natural outbreaks (which complicates Phase 3 efficacy trials), ethical constraints on human challenge studies, and limited commercial incentives due to the disease’s low global incidence. Consequently, most vaccines are stockpiled for emergency or military use rather than public distribution. To address these challenges, the WHO has developed a Target Product Profile (TPP) for plague vaccines. The TPP defines the minimum and preferred characteristics of future vaccines, including safety, efficacy, dosing, and storage requirements, to guide developers toward products that are suitable for outbreak response and equitable access. This framework supports SDG 3: Good Health and Well-being, by promoting the development of safe, effective, and affordable vaccines for all populations, including those in low-resource settings (Fig. 1).

### Vaccination against Yersiniosis

For *Y. enterocolitica, Y. pseudotuberculosis*, and *Y. ruckeri*, strain heterogeneity, antigenic diversity and host-specific pathogenicity have complicated subunit vaccine design (Caugant et al. 1989, Tinsley et al. 2011, Tadesse et al. 2013). Rational attenuation strategies and host-adapted models are essential for advancing this approach. Current efforts focus on identifying conserved surface-exposed antigens and virulence factors with cross-protective potential (Casutt-Meyer et al. 2010, Batzilla et al. 2011). Advances in reverse vaccinology and proteomics are hastening this discovery process (Strong et al. 2011, Heine et al. 2019, Yang et al. 2021).

In particular, *Yersinia ruckeri* vaccine development has accelerated due to its economic impact on aquaculture. Recent innovations include immersion and oral vaccines tailored for early life stages of fish, offering improved onset of immunity, longer protection, and reduced handling stress (Ghosh et al. 2016, Yang et al. 2021). Advances in antigen discovery, particularly outer membrane proteins such as OmpA, OmpF, and heat shock proteins, have enabled the design of vaccines with cross-serotype efficacy, addressing the challenge of strain variability (Ormsby et al. 2019, Chukwu-Osazuwa et al. 2022). These strategies align with sustainable aquaculture practices and SDG 12: Responsible Consumption and Production, by reducing reliance on antibiotics, minimizing environmental impact, and supporting resilient food systems (Fig. 1).

### Future perspectives surrounding the *Yersinia* vaccinology landscape

These developments underscore a dynamic and evolving landscape in *Yersinia* vaccinology. Future efforts will benefit from integrating structural vaccinology, systems immunology, and host-pathogen interaction studies to refine antigen selection, optimise delivery platforms, and tailor vaccines to diverse hosts and epidemiological contexts. Embedding sustainability principles into vaccine design and deployment, whether through reduced ecological impact, improved accessibility, or alignment with One Health frameworks, will be critical to ensuring long-term global health resilience.

## Probiotics as an alternative for *Y. ruckeri* infection

Probiotics represent a promising alternative to antibiotics and vaccines in aquaculture, offering an environmentally sustainable approach to disease management (Fig.  1). Beneficial bacteria, including established probiotic genera such as *Bacillus, Aeromonas* and *Lactobacillus*, are well documented in their ability to protect fish against *Yersinia ruckeri* and other major pathogens. Their cellular components, including cell wall proteins, outer membrane proteins and lipopolysaccharides, contribute to reduced mortality by stimulating host immunity, producing antimicrobial compounds and competing with pathogens for nutrients, energy sources and adhesion sites (Raida et al. 2003, Abbass et al. 2010). Alongside these firmly validated bacterial probiotics, interest is growing in yeast-based probiotics whose beta-glucan rich cell walls can enhance immune function. Species such as *Saccharomyces cerevisiae, Debaryomyces hansenii, Candida zeylanoides* and *Rhodotorula mucilaginosa* are emerging as promising candidates (*Çayli Bektaş* et al. *2024*), although they remain less thoroughly characterised and less directly linked to *Y. ruckeri* protection than bacterial probiotics.

Recent research has increasingly focused on leveraging fish-associated microbiomes to improve aquaculture productivity and sustainability. Dysbiosis within the microbiome has been linked to disease susceptibility, whereas targeted modulation through probiotics or dietary interventions can stabilise gut microbial communities and enhance pathogen resistance (Ingerslev et al. 2014, Rasmussen et al. 2022). Despite these advances, the definition of a “healthy” microbiome remains poorly characterised for most fish species. This knowledge gap is further complicated by the influence of environmental variables—such as salinity, pH, temperature, and ionic composition—on microbiome dynamics. Consequently, optimising these parameters is essential for maintaining microbiomes that support both sustainability and productivity in aquaculture systems.

Future research should prioritise multi-omics approaches to define core microbiomes across diverse fish species and production environments. Longitudinal studies are needed to elucidate microbiome succession during different life stages and under varying environmental conditions. Additionally, mechanistic investigations into host–microbe interactions will inform the development of next-generation probiotics tailored to specific species and farming systems. Integrating microbiome engineering with precision aquaculture technologies could enable real-time monitoring and adaptive management of microbial communities, ultimately advancing disease prevention and improving overall system resilience.

## Concluding remarks: from concept to clinical reality

Decades of intensive research have generated a remarkably broad repertoire of innovative therapeutic ideas for combating *Yersinia* infections, ranging from anti-virulence compounds and secretion-system inhibitors to host-directed therapies, phage formulations, metabolic targeting strategies, and diverse vaccine candidates (Fig. 1 and Table 1). However, none of these approaches has yet progressed to licensed application in clinical medicine or routine public-health practice. This striking disconnect between laboratory innovation and real-world implementation reflects a series of persistent bottlenecks encountered throughout the translational pathway.

**Figure 2:**
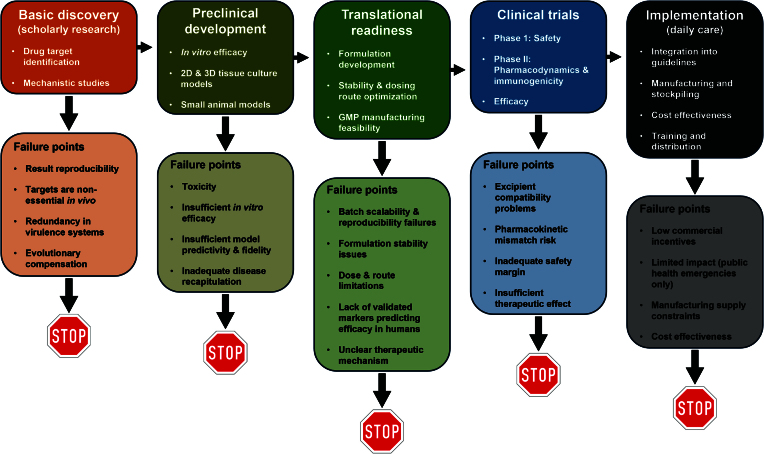
Translational bottlenecks limiting the progression of anti-*Yersinia* interventions from conceptual discovery to real-world implementation. Shown is a summary of the major stages in the development pipeline for vaccines, anti-virulence agents, host-directed therapies, phage-based approaches, and other countermeasures against *Yersinia* infections. Beginning with basic scholarly discovery, the pathway progresses through preclinical development, translational readiness, and clinical trials before reaching implementation in routine practice. At each stage, characteristic failure points impede progress: non-essential or redundant virulence targets in early discovery; toxicity and limited predictive power of *in vitro* and small-animal models in preclinical testing; lack of validated correlates of protection and challenges in formulation and manufacturing feasibility during translational preparation; restricted trial feasibility, low commercial incentives, and regulatory barriers during clinical evaluation; and, finally, limited adoption, cost-effectiveness constraints, and dependence on emergency-only use during implementation. Collectively, these barriers illustrate why many promising anti-*Yersinia* strategies fail to advance to licensed, widely deployable interventions.

A major barrier arises as early as the discovery and preclinical phases: compounds that show strong *in vitro* activity frequently fail to deliver meaningful efficacy *in vivo* (Fig.  2). This disconnect is often driven by unfavourable pharmacokinetic profiles, toxicity, or inadequate target accessibility; limitations that undermine the potential of otherwise promising anti-virulence candidates. Such pharmacological constraints are particularly critical for agents designed to act at the host–pathogen interface, where achieving sufficiently high local concentrations is essential for therapeutic impact. Together, these limitations highlight that the host–pathogen interface constitutes a complex ecosystem, shaped by dynamic and interdependent physiological processes. Our understanding of how these parameters converge to shift the system from equilibrium into an infectious disease state remains rudimentary (*Burmeister* et al. *2021*).

A further impediment arises from the lack of validated correlates of protection, which are defined and measurable immune markers that reliably predict protection against plague in humans (Fig. 2). Since such biomarkers have not been established for *Yersinia* infections, developers cannot use immune readouts to infer the likely effectiveness of vaccines or host-directed therapies, and regulators cannot rely on surrogate endpoints, which are immunological measurements that normally stand in for true clinical protection. This leaves promising candidates without the evidentiary foundation needed to advance beyond early-stage development.

Compounding these difficulties is the stringent regulatory framework governing work with *Yersinia pestis* (Fig. 2). In the United States, *Y. pestis* is designated a Tier 1 Select Agent, a category reserved for pathogens deemed to pose the highest potential risk to public health or national security. This designation mandates enhanced biosafety and biosecurity controls, including controlled access, comprehensive personnel screening, detailed inventory management, and strict federal reporting requirements. In Europe, *Y. pestis* is classified as a Risk Group 3 biological agent under the EU Biological Agents Directive 2000/54/EC and corresponding national legislation, requiring full BSL-3 containment, specialised engineering controls, and rigorous occupational-safety procedures. Although the two systems differ in terminology and legislative basis, both impose substantial containment and administrative burdens that limit the number of laboratories authorised to conduct high-risk experiments. These constraints, while essential for biosafety, significantly slow iterative optimisation of candidate vaccines, anti-virulence compounds, phage therapeutics, and other interventions.

Economic considerations further impede progress (Fig.  2). Plague and severe yersiniosis are low-incidence diseases, offering limited commercial incentives for industry investment in vaccines or therapeutic development. Even in aquaculture, where *Y. ruckeri* infections impose substantial economic burdens, the development of new interventions remains constrained by stringent regulatory requirements, high production and validation costs, and narrow profit margins. This persistent misalignment between public health value and market viability has slowed innovation across multiple otherwise promising technological domains and extends well beyond the *Yersinia* field (Niño-Vega et al. et al.,  2025).

Scientific hurdles also play a decisive role (Fig. 2). Antigenic variability, particularly within LcrV, complicates the design of cross-protective vaccines, as polymorphisms can undermine antibody recognition. Phage-based therapies face the rapid emergence of bacterial resistance through receptor modification, often necessitating multi-phage cocktails to maintain efficacy. Similarly, while anti-virulence strategies offer an appealing means of disarming *Yersinia* without exerting classical selective pressure, inhibition of a single virulence activity may theoretically be compensated by the activation of alternative pathways. The idea is termed evolutionary compensation (*Vale* et al. *2016*) and could arise through regulatory rewiring, metabolic reprogramming, or broader shifts in physiological potential, with the possibility of even facilitating niche expansion under certain conditions. These concerns are largely conceptual at present, as the extent to which such compensatory behaviour occurs has not been systematically investigated. Furthermore, any such effects are likely to be highly context dependent: pathogen behaviour observed *in vitro* may differ substantially from that *in vivo*, where metabolic constraints, immune pressures, and environmental stresses vary dramatically. Together, these considerations raise important theoretical questions about the durability and robustness of anti-virulence strategies in real-world infection settings.

Taken together, these constraints highlight why so many theoretically compelling anti-*Yersinia* approaches remain confined to the conceptual or early preclinical stages. Overcoming these obstacles will require a more deliberate integration of mechanistic innovation with translational feasibility: early pharmacokinetic optimisation, development of standardised immunological endpoints, regulatory strategies adapted to work within select-agent frameworks, sustainable economic models for low-incidence diseases, and combination therapies resilient to evolutionary escape. Through coordinated scientific, regulatory, and policy efforts, the field can reposition itself to translate conceptual advances into deployable tools that meaningfully enhance preparedness and response to *Yersinia* infections.

## Data Availability

As a review article, there are no data to make available.
